# Targeting natural killer cells: from basic biology to clinical application in hematologic malignancies

**DOI:** 10.1186/s40164-024-00481-y

**Published:** 2024-02-23

**Authors:** Juanjuan Shang, Shunfeng Hu, Xin Wang

**Affiliations:** 1 Department of Hematology, Shandong Provincial Hospital, Shandong University, No.324, Jingwu Road, Jinan, 250021 Shandong China; 2 Department of Hematology, Shandong Provincial Hospital Affiliated to Shandong First Medical University, Jinan, 250021 Shandong China; 3 Taishan Scholars Program of Shandong Province, Jinan, 250021 Shandong China; 4 Branch of National Clinical Research Center for Hematologic Diseases, Jinan, 250021 Shandong China; 5 National Clinical Research Center for Hematologic Diseases, the First Affiliated Hospital of Soochow University, Suzhou, 251006 China

**Keywords:** NK cells, Hematologic malignancies, Metabolism, Immunotherapy, Clinical trials

## Abstract

Natural killer (NK) cell belongs to innate lymphoid cell family that contributes to host immunosurveillance and defense without pre-immunization. Emerging studies have sought to understand the underlying mechanism behind NK cell dysfunction in tumor environments, and provide numerous novel therapeutic targets for tumor treatment. Strategies to enhance functional activities of NK cell have exhibited promising efficacy and favorable tolerance in clinical treatment of tumor patients, such as immune checkpoint blockade (ICB), chimeric antigen receptor NK (CAR-NK) cell, and bi/trispecific killer cell engager (BiKE/TriKE). Immunotherapy targeting NK cell provides remarkable advantages compared to T cell therapy, including a decreased rate of graft versus-host disease (GvHD) and neurotoxicity. Nevertheless, advanced details on how to support the maintenance and function of NK cell to obtain better response rate and longer duration still remain to be elucidated. This review systematically summarizes the profound role of NK cells in tumor development, highlights up-to-date advances and current challenges of therapy targeting NK cell in the clinical treatment of hematologic malignancies.

## Introduction

Recently, a remarkable potential has been demonstrated in immunotherapy for treating tumors, including adoptive cell therapy, CAR, ICB, and bi/trispecific immune cell engagers. Therapeutic strategies targeted immune cells have changed traditional tumor therapeutic regimen, particularly chimeric antigen receptor T (CAR-T) cells that could confer higher specificity and affinity to T cells [1]. Established efficacy profiles of utilizing T cells has led to the emerging impetus to develop T cell-targeted tumor immunotherapy and promoted extensive clinical investigations [2]. However, intolerable toxicities including GvHD and neurotoxicity [3] greatly limit clinical application of T cell therapeutic strategies. Therefore, emerging studies have energized a shift of focus towards clinical utility of different immune cells, especially NK cells.

NK cell, identified with absence of surface T cell receptor (TCR) and related cluster of differentiation (CD) 3 molecule, acts as tumor suppressors and contribute to host immune surveillance and defense without pre-immunization [4]. Under healthy conditions, NK cells are damped to protect normal cells from damage due to the interaction between human leukocyte antigen (HLA) of normal nucleated cell and killer cell immunoglobulin-like receptors (KIRs) [5, 6]. NK cells are rapidly activated and then exert cell-killing activity once encounter abnormal cells with decreased expression of HLA molecule, which contribute to the clearance of abnormal cells and the persistence of organism homeostasis [6]. NK cells perform their cytotoxic activity mainly through two ways: I) directly contact with target cells and release perforins and enzymes associated to cell lysis [7]. II) produce immunomodulatory factors including an array of cytokines and chemokines, mainly interferon-γ (IFN-γ), to modulate adaptive immunity and participate in other associated pathways [8, 9].

NK cell immune function is restricted in tumors, which suggests valuable therapeutic targets for tumor treatment. The first application of NK cells in tumor therapy could be traced back to 1985, precleared autologous NK cells were transferred to patients with metastatic cancer and obviously decreased tumor volume (more than 50 percent) [10]. Subsequent strategies for boosting NK cell function including being modified with CAR structures or engagers, and blocking inhibitory receptors with ICB, which attract more and more attention as encouraging anticancer therapeutics. Here, we review biological function and dysfunctional roles of NK cells in hematologic tumors, recent advances and current challenges of NK cell-targeted clinical applications, which might offer novel therapeutic strategies for patients with hematologic malignancies.

## The dysfunction of NK cells in hematologic malignancies

### Decreased function of NK cells in tumor microenvironment

#### Abnormal features of receptors on NK cells

**Fig. 1:**
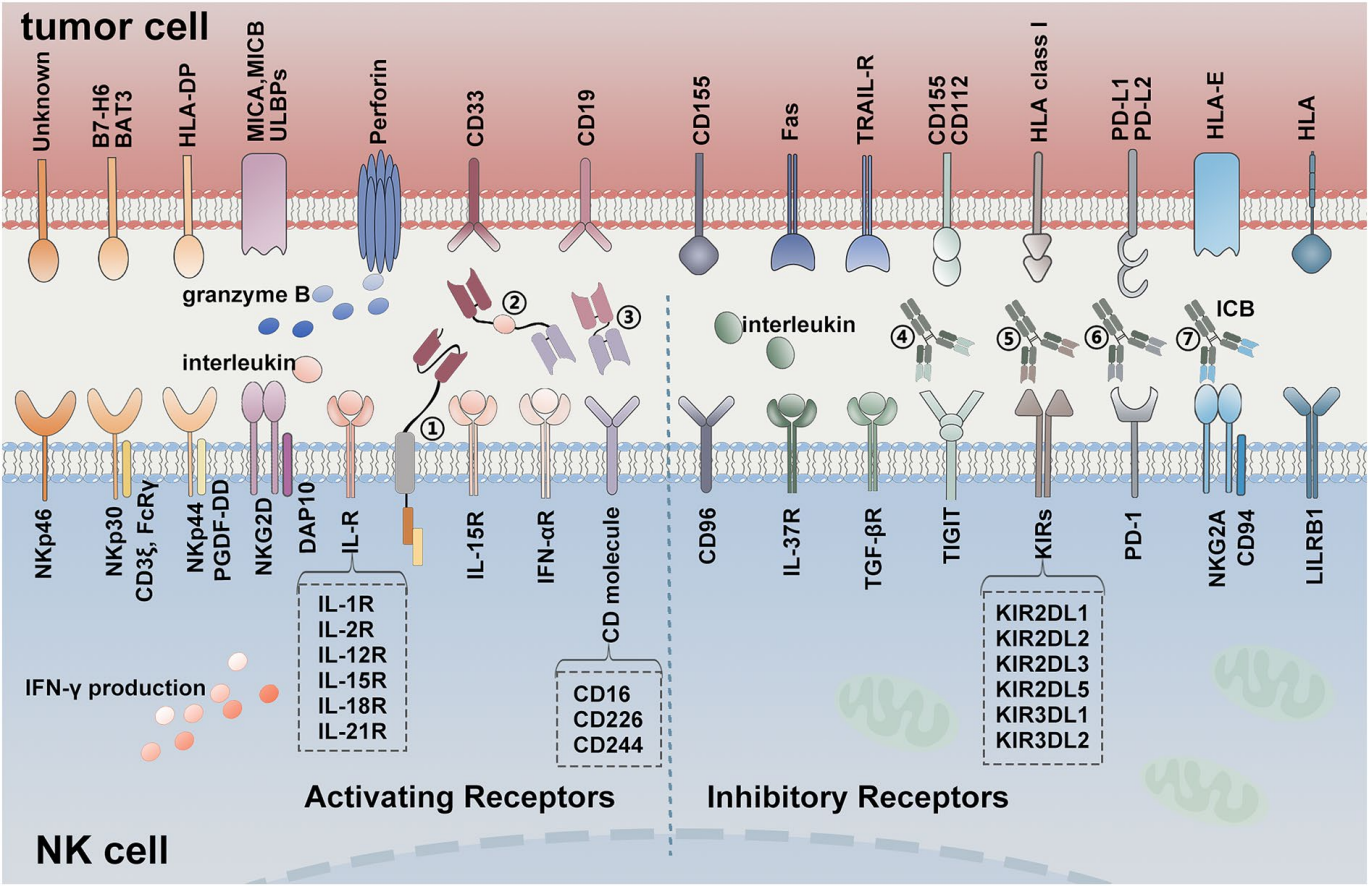
General view of surface receptors between NK cell and tumor cell. The anti-tumor functions of NK cell depend on a suite of activating and inhibitory receptors which combined to ligands on tumor cell surface or regulatory cytokines. Dramatical changes of the interaction lead to NK cell dysfunction and then cause tumor escape and progression. Novel agents targeting these special interactions have been explored to augment NK cell-mediated killing effect and recruit them to boost anti-tumor immunity. (**1**) CD33 CAR molecule; (**2**) CD-16/IL-15/CD33 TriKE; (**3**) Anti-CD16/CD19 BiKE; (**4**) Anti-TIGIT mAb, such as Tiragolumab; (**5**) Anti-KIR mAb, such as Lirilumab; (**6**) Anti-PD-1 mAb, such as Pembrolizumab; (**7**) Anti-NKG2A mAb, such as Monalizumab. BAT-3, HLA-B associated transcript 3; BiKE, bispecific killer cell engager; B7-H6, B7 homolog 6; CAR, chimeric antigen receptor; CD, cluster of differentiation; DAP10, DNAX-activation protein 10; Fas, factor related apoptosis; FcRγ, fragment crystallizable receptor γ; HLA, human leukocyte antigen; ICB, immune checkpoint blockade; IFN-αR, interferon-α receptor; IFN-γ, interferon-γ; IL-R, interleukin receptor; LILRB1, leukocyte immunoglobulin-like receptor B 1; mAb, monoclonal antibody; MICA, human MHC-I chain-related A; MICB, human MHC-I chain-related B; NKG2A, natural killer group 2 member A; NKG2D, natural killer group 2 member D; NKp30, natural killer receptor protein 30; NKp44, natural killer receptor protein 44; NKp46, natural killer receptor protein 46; KIRs, killer cell Ig-like receptors; PD-1, programmed cell death 1; PD-L1, programmed cell death ligand 1; PD-L2, programmed cell death ligand 2; PGDF-DD, platelet derived growth factor DD; TGFβR, transforming growth factor-β receptor; TIGIT, T cell Ig and ITIM domain; TRAIL-R, TNF-related apoptosis inducing ligand-receptor; TriKE, trispecific killer cell engager; ULBPs, ULl6 binding proteins

Activating, inhibitory and co-stimulatory receptors are needed for NK cell functions, which display obvious abnormalities in tumors. Figure 1 shows classical surface receptors of NK cell. Natural killer group 2 member D (NKG2D) receptor and natural cytotoxicity receptor (NCR) are two prominently stimulatory receptors contributing to NK cell activation [11, 12], decreased expression of them leads to NK cells dysfunction in tumor environment [13]. Previous studies have shown dramatical changes of surface epitopes on tumor cells, such as NKG2D ligand (NKG2DL)-negative could help tumor cells to escape. Increasing NKG2DL levels of tumor cells through genetically or pharmacologically inhibiting poly-ADP-ribose polymerase 1 (PARP1) could suppress leukemogenesis in patient-derived xenotransplant models [14].

Published studies have suggested that the impairment of NK cytolytic function derived in part from reduced specific molecules on tumor cell surface. Loss of the mismatched HLA in the genome of the leukemia impaired NK cell-mediated response, representing a vital immune escape mechanism of leukemia relapse after allogeneic hematopoietic cell transplantation [15, 16]. Tumor cells could downregulate the expression of stimulatory ligand through several strategies, including DNA methylation [17], expression pattern alteration of related gene [18, 19] and self-shedding from the cell surface [20]. For example, major histocompatibility complex (MHC)-I chain related protein A (MICA) molecules expressed on malignant cell activated NK cell via binding to NKG2D, while soluble MICA releasing from malignant cell surface impaired NKG2D and facilitated escape of them from immunosurveillance [21, 22]. Inhibition of MICA shedding via antibodies or vaccines were demonstrated to promote anti-tumor immunity of NK cells, providing rationales for exploring novel clinical applications targeting NK cell receptors [23–25]. Exosomes, a population of vesicles in biological fluids, were demonstrated to downregulate the expression of NKG2D by inducing its internalization from NK cell surface in microenvironment of leukemia and multiple myeloma (MM) [26–28]. Altogether, these findings provide a novel insight that related ligand levels of tumor cells may become a prognostic index, targeting these specific interactions will strengthen the killing effect of NK cells and improve therapeutic outcomes for hematologic malignancies in the future.

Inhibitory receptors on NK cell surface constrain killing capacity of them, which are indispensable for self-tolerance [29]. Natural killer group 2 member A (NKG2A), KIRs family, T cell immunoglobulin and immunoreceptor tyrosine-based inhibitory motif domain (TIGIT) and programmed cell death protein 1 (PD-1) are representative inhibitory receptors. Among them, NKG2A-CD94 complex on NK cell surface transduces inhibitory signals through binding to HLA-E of tumor cells, which rendered the lytic activity of NK cell impaired [13, 30]. Up-regulation of HLA-E was found in some hematologic neoplasms such as chronic lymphocytic leukemia (CLL) and acute myeloid leukemia (AML), indicating a novel strategy to restore cytotoxic ability of NK cell via blocking NKG2A on cell surface [31]. TIGIT expression was demonstrated to be tightly restricted in lymphocytes including T cell subsets and NK cells [32]. Increasing evidence has demonstrated that TIGIT was highly expressed on tumor-infiltrating NK cells in hematological malignancies, such as AML, resulting in tumor progression and poor outcomes [33]. Zhang et al. found that TIGIT expression on tumor-infiltrating NK cells was associated with functional exhaustion of NK cells, and blockade of TIGIT via monoclonal antibodies reversed the exhaustion of anti-tumor immunity [34]. Furthermore, a marked increase level of PD-1 was detected in tumor-infiltrating NK cells of hematologic malignancies [35–38]. PD-1^+^ NK cells were proved to have a tendency to exhaust, characterized by reduced proliferative capability and impaired cytotoxicity in tumor microenvironment (TME) [39]. Previous studies focusing on PD-1 and its ligands have found that blockade of the interaction can restore IFN-γ-producing function of NK cells. Besides, soluble programmed cell death ligand 1 (PD-L1) in serum were found to be related to adverse prognosis in lymphoma [40, 41]. Taken together, interactions between inhibitory receptors and the ligands inhibit cytokines release and anti-tumor cytotoxicity of NK cell, which causes immune escape of tumor development. Further detailed research is still needed to explore the intricate network between inhibitory receptors and the ligand in TME of hematologic malignancies.

#### Disruption of killing pathways in NK cells

Activated NK cells take anti-tumor effect mainly through direct or indirect mode. In direct killing mode, immunological synapses forming between tumor cell and NK cell led to release of lysosomes-like molecules such as granzymes and perforins, the perforation of tumor cell membrane, and the induction caspase-dependent or -independent apoptosis [7, 42]. Tumor initiation and metastasis were suppressed by NK cell released perforins, growth of tumor cells was proved to be inhibited by perforin-dependent cytotoxicity of NK cells in mice [43]. It was confirmed that lacking perforin in NK cells contributed to failure in restraining the metastasis of malignant cells to lung [44]. Indirect way mainly refers to the effect of cytotoxic and regulatory cytokines. Factors secreted by NK cells including cytokines, chemokines (such as chemokine CC-chemokine ligand (CCL) 3, CCL4, CCL5), adenosine, and growth factors, exert functions of regulating innate and adaptive immunoreactions [45]. For example, NK cell could facilitate the maturation of DCs through IFN-γ and tumor necrosis factor (TNF), and the initiation of CD4^+^ T helper cells in the inflamed lymph node also relied on IFN-γ [46].

### Metabolic alterations of TME impair NK cell functions

Metabolic reprogramming has been widely revealed in cancer cells with the appearance of increased glycolysis, lipid synthesis and amino acids catabolism, which not only serves as crucial determinant in signal pathways for sustaining tumorigenesis, but also has profound implication to immunocytes [47, 48]. Immune cells including NK cells have been subsequently found in engagement in metabolic manipulation due to competition for fuels with malignant cells in TME. Depletion of nutrients, aberrant accumulation of toxic metabolites and intermediates in TME influence NK cell proliferation and effector function. Clinical applications of targeting tumor metabolism have emerged and achieved remarkable progresses over the past decades, such as using metabolomics-based biomarker for early diagnosis and therapeutic approaches that aim at metabolic enzymes or metabolites [49, 50].

#### Glucose deficiency

Glucose is a widely described poor fuel in TME attributing to Warburg effect, a main character of tumor cells that avidly utilize and convert glucose to lactate even oxygen is sufficient [51–53]. Decreased concentration of extracellular glucose and following reduced glycolysis and oxidative phosphorylation (OXPHOS) attenuate cytotoxic ability of NK cells, for example, by reducing IFN-γ and Fas ligand levels [54]. In addition, rapid growth of malignant cell consumes abundant glucose and creates a high-lactate microenvironment [55, 56]. Studies pointed out gradual loss of IFN-γ production of NK cells during tumor progress, partly attributing to low pH level and accumulation of lactate in TME. Pathophysiological concentrations of lactate could affect levels of nuclear factor of activated T cell (NFAT) in NK cells, causing diminished IFN-γ production [57]. In lymphoid organs the quantity of NK cell and IFN-γ level could be recovered when systemic alkalified by oral delivery of bicarbonate [58]. Similar to the mechanism of elevated lactate in solid tumor environment, tumor cells of hematologic malignancies take up a large amount of glucose and produce lactate via lactate dehydrogenase A (LDHA) due to genetic changes and tumor hypoxia. Lactic acids accumulate in cells and then are exported through monocarboxylate transporters (MCTs) on cell membrane, contributing to an acidic TME [59]. Indeed, this provides us a novel idea for functional reversal of NK cells, as to control balance of glucose levels via crucial targets such as LDHA and MCTs. Reduction of LDHA function by small interfering RNA (siRNA) or a small-molecule inhibitor FX11 was proved to lead to tumor regression [60]. Selective MCTs inhibitors could decrease intracellular pH and impair the proliferation of malignant cells such as leukemia cells, which were expected to become a promising adjunct in tumor treatment strategy [61–63]. AZD3965, an orally bioavailable MCT1 inhibitor, has been currently under phase I clinical trial in patients with advanced tumors including lymphoma [NCT01791595] [64]. These findings indicated that targeting glucose metabolism may be an opportunity for novel treatment strategies in hematologic malignancies.

#### Aberrant lipid accumulation

Malignant cells increase de novo fatty acid synthesis to provide enough energy for anabolic and signaling pathways, leading more aberrant metabolites accumulation such as short-chain fatty acids, which have been confirmed to skew immunocytes towards immunosuppressive phenotypes [65]. Lipid metabolism associated transcriptional reprogramming of NK cells and abnormality signaling mediated immunosuppressive microenvironment were detected in tumors such as in aggressive B cell lymphoma [66, 67]. Cellular metabolism and effector responses of NK cells are potently suppressed when exposed to fatty acids, which mainly due to a significant rewiring of lipid metabolic pathways [66, 68]. Transcriptional and single cell analysis suggested significant upregulation of lipid and glycerol uptake-related genes expression of NK cells when exposed to lipid-enriched TME, leading to impairment and dysfunction of NK cells [69, 70]. Higher cholesterol concentration is also prone to facilitate the expression of ICBs of immune cells, resulting in lower proliferation and cytotoxicity capacity. Metabolites and varieties of physiological substances of cholesterols also contribute to the immunological landscape. For instance, accumulation of 22-hydroxycholesterol can recruit CD11b^high^Gr1^high^ neutrophils, an important immunosuppressive population in the TME [71]. Adrenal cortex hormones, transformed from cholesterols, can significantly suppress the proliferation and activity of many immune cells including NK cells [72, 73]. NK cells were found to neutralize toxicity of increased lipid levels in TME through upregulating peroxisome proliferator-activated receptor (PPAR) related signaling pathway to improve synthesis of lipid. Previous studies found that employing a specific agonist Rosiglitazone could stimulate PPAR-γ and thus recovered partial function of NK cells [66, 74]. Besides directly alters metabolic pathways of NK cell, the aberrant accumulation of lipid metabolites also contributes to immunosuppressive phenotypes by acting on immunoregulatory cells in TME such as DC and bone marrow derived suppressor cell (MDSC), which create an immunosuppressive microenvironment and impair NK cell activity [75].

#### Amino acids starvation and enzyme abnormality

Aberrant profile of amino acids metabolism has been found in diverse tumors. For instance, acute lymphoblastic leukemia (ALL) cells need an exogenous source of asparagine, and exhausting the amino acid from blood has been a prominent component of ALL chemotherapy for decades [76]. Solute carrier family 1 member 1 (SLC1A1), a soluble ectopic transporter on tumor cell membrane increased cellular glutamine uptake. Cell proliferation and tumor growth were both accelerated by this glutamine addiction [77]. When glutamine became deficient or L-amino acid transport was systematically blocked, NK cell growth response was impaired rapidly by regulating c-myc protein levels [78]. Arginine (Arg)-starved NK cells show weak viability, decreased NKp46 and NKp30 levels, and reduced intracellular production of IFN-γ [79]. Researchers proved MDSCs expressed arginase I and AML blasts secreted arginase II, both could specifically agitate M2 phenotypes in surrounding monocytes to inhibit NK cell immune response [80]. In addition, Arginase I can convert Arg to ornithine and then limit the proliferation of NK cells. CB-1158, a potent arginase I inhibitor with high affinity, partially restores NK cell function and blunts AML cell immune escape by inhibiting Arg depletion [81]. One of the obstacles is that few amino acids are only confined to tumor cells, systemic blockades raise the likelihood of toxicity to normal cells thus affect organs such as brain and heart [76]. A more intense knowledge of amino acid utilization in both tumor cell and NK cell is still required to develop tumor therapies targeting amino acid metabolism.

#### Reactive oxygen species (ROS) cytotoxicity

ROS is generally deemed to by-product of oxygen consumption and cell metabolism formed by the partial reduction of molecular oxygen [82]. ROS is certified to be related to tumor initiation and progression as it activates pro-tumorigenic signaling, drives genetic instability and DNA damage, enhances cell proliferation and survival [83]. Tumor cells produce much more ROS than normal cells which damage killing effect of NK cells. For instance, chronic myelogenous leukemia (CML) cells can motivate nicotinamide adenine dinucleotide phosphate (NADPH) oxidase to generate and then paracrine ROS, contributing to the dysfunction of NK cells [84]. Johan Aurelius et al. discovered an original mechanism of NK cells apoptosis depend on triggered PARP1, demonstrating that oxygen radicals could cause NK cells to undergo apoptosis [85]. Previous clinical trial investigated the effect of histamine dihydrochloride, a NADPH oxidase inhibitor, showed notable improvement of leukemia-free survival and AML relapse prevention [86]. Moreover, studies have confirmed that the levels of ROS and the activity of anti-oxidant enzymes are typically increased in drug resistant tumor cells, indicating ROS may become an attractive target for overcoming chemotherapy resistance. Inhibitors targeting ROS production pathway, such as critical redox-regulating enzymes, show bright prospects in immune recovery of NK cells [87].

### Other immunosuppressive factors

#### Hypoxia in TME

Hypoxia, attributed to insufficient oxygen supply and rapid tumor growth, has been demonstrated to play a vital role in tumor progression and resistance to therapy. Similar to solid tumors, hypoxia condition was reported to be important for tumor neovasculogenesis, metastasis and drug resistance in hematologic malignancies [88, 89]. Published studies have revealed that hypoxia reduced the ability of NK cells to release cytokines, such as granulocyte–macrophage colony stimulating factor (GM-CSF), IFN-γ and TNF-α, and decreased the expression of granzyme B and degranulation marker CD107a, thus facilitated tumor immune escape [90]. Teng et al. found that the dysfunction of NK cells under hypoxic condition may be attributed to the activation of protein tyrosine phosphatase SHP-1. Knocking down SHP-1 or using a specific inhibitor TPI-1 was able to partially restore NK cell cytotoxicity under hypoxia [91]. These findings pointed out targeted inhibitors of hypoxia-activated molecules might provide promising therapeutic efficacy for patients with specific profile of hypoxia biomarkers.

#### Chemotactic environment abnormality

NK cells develop from bone marrow parenchyma and traffic into various tissues to perform specific functions [92]. Previous studies have noted that chemotactic components abnormality in TME could disturb the recruiting and function of NK cells. Dysregulation of C-X-C chemokine receptor 3 (CXCR3) and CXCR4 chemokine receptor-ligand axis was found to induce defective migration and retention of NK cells in MM [93]. Under the influence of CXCR1 and CXCR2 receptor agonists and other chemotactic factors produced by tumors, neutrophils and MDSCs extruded neutrophil extracellular traps, which wrapped tumor cells and shield them from cytotoxicity mediated by NK cells [94]. Beyond the impact of NK cell trafficking, complex receptor-ligand mode in TME also leads to downregulation of cytotoxicity [95]. For example, IFN-γ secretion of NK cells could be activated through the CXCL10/CXCR3 axis when interferon regulatory factor 1 increased [96]. Hence, altering the expression levels of chemotactic factors in TME will help enhance the infiltration and activation of NK cells, possible further strengthen the therapeutic effect of NK cells.

#### Tumor-secreted immunosuppressive factors

A variety of immunosuppressive factors containing anti-inflammatory cytokines and specific enzymes have been elucidated to play a role in the immune escape of tumor cells in recent studies [17]. In many hematologic malignancies such as AML and CLL, anti-inflammatory cytokines including IL-4, IL-10, and TGF-β produced by tumor cells could render them less immunogenic, thus caused immune escape [97, 98]. Interestingly, Wang et al. found that effector function of bone marrow-derived NK cells in AML patients were impaired by higher levels of TGF-β1 in TME. Galunisertib, TGF-β1 pathway inhibitor, could significantly restore the cytotoxicity and anti-tumor activity of NK cells, providing a potential therapeutic method to improve outcomes in AML patients [99]. Besides, there were hypothetical mechanism that the suppression of proinflammatory growth factors such as granulocyte colony-stimulating factor (G-CSF) and IL-1β also played a role in immune escape [15]. Some immunomodulatory enzymes and metabolites of catalytic reaction were found to be associated with immunosuppressive TME, further impaired NK cell function. Indoleamine 2,3-dioxygenase (IDO) expression was reported in both bone marrow and peripheral blood AML blasts, displayed anti-tumor effect by inducing Treg cells and impairing IFN-γ production of T and NK cells [100, 101]. These results highlighted that the measurement of such specific enzymes may offer utility as prospective prognostic markers, and targeted inhibitors may hold promise for the treatment of tumors.

## Targeting NK cells in hematologic malignancies

**Fig. 2:**
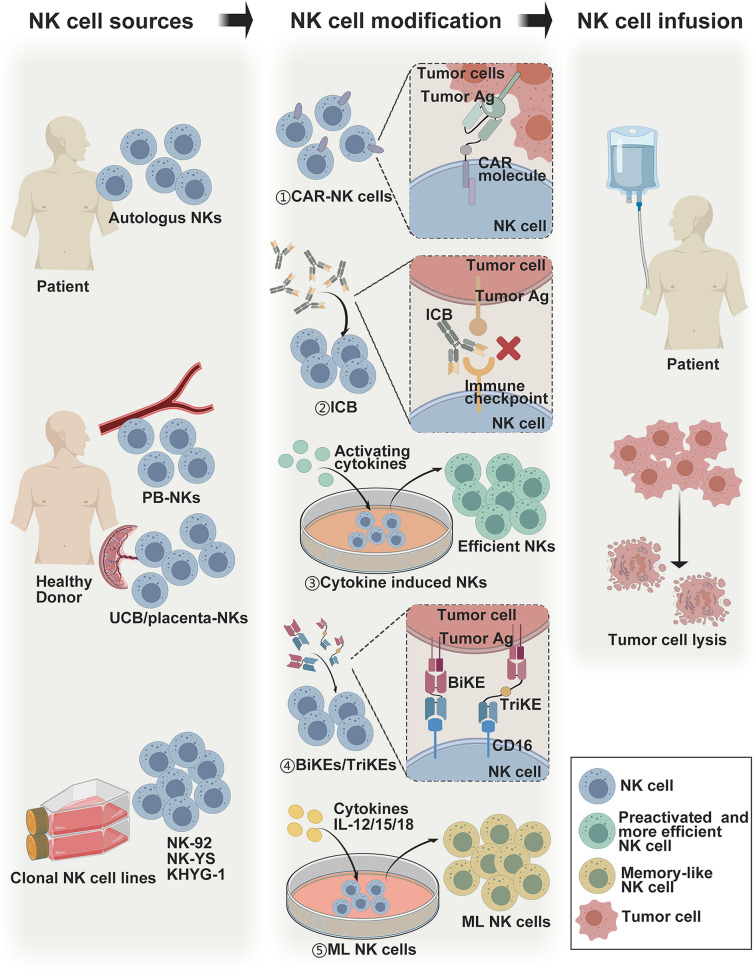
General view of NK cell-based immunotherapies. (**1**) A number of NK cell sources were explored for infusion in adoptive NK cell therapy, including autologous NK cell from patients themselves, peripheral blood or umbilical cord blood and placenta-derived NK cell from healthy donors, and clonal NK cell lines such as NK-92, NK-YS, KHYG-1. (**2**) Strategies targeting to enhance anti-tumor activity of NK cell including employing CAR structures, using immune checkpoint blockades to block inhibitory receptors on NK cell surfaces, augmenting NK cells by cytokines, applying BiKEs and TriKEs, and inducing memory-like NK cells. (**3**) Biologically active agents or the “activated” NK cells were then infused to patients, causing lysis of tumor cells and further improving the clinical outcome. Ag, antigen; BiKE, bispecific killer cell engager; CAR, chimeric antigen receptor; CD, cluster of differentiation; IL, interleukin; ICB, immune checkpoint blockade; ML-NKs, memory-like natural killer cells; NKs, natural killer cells; PB, peripheral blood; TriKE, trispecific killer cell engager; UCB, umbilical cord blood

Flourishing immunotherapy has become the fourth tumor treatment option after chemotherapy, radiotherapy and surgery. With the development of T cell-based immunotherapy including CAR-T, non-specific immunity has also gained attention with better efficacy and fewer side effects. Here follows NK cell-based immunotherapies being gradually explored for hematologic malignancies, of which general view is recapitulated in Fig. 2.

### The source of NK cells for adoptive immunotherapy

Adoptive infusion of NK cells has overcome uncontrolled acute GvHD reaction, the principal barrier of adoptive T cell therapy [102]. The first exploited sources for adoptive NK cell therapy were autologous NK cell as early as 1985. Metastatic cancer patients who had failed in standard therapy were treated with 1.8 to 18.4 × 10^10^ autologous NK cells, observing objective regression (more than 50 percent of tumor volume) in 11 of the 25 patients [10]. Hareth Nahi et al. demonstrated the feasibility of infusing autologous NK cells in MM patients [103]. Another phase II clinical trial of adoptive transfer of haploidentical NK cells found no decrease of the cumulative incidence of relapse and no improvement of overall survival (OS) in AML patients, which mainly attributed to the limited persistence of alloreactive donor NK cells [104]. Further research discovered that using activating factors such as IL-2 could assist augmentation of NK therapeutic efficacy. However, IL-2 also added toxicity and complication including elevation of creatinine and bilirubin levels, oliguria, hypotension at the same time [105, 106]. Human umbilical cord blood (UCB) and placenta are rich sources for cytotoxic CD56^+^ NK cell which has high lytic capabilities. It is estimated that around 30% of lymphoid populations in UCB are NK cells, which tend to be younger and have a stronger proliferation potential [107]. Harry Dolstra et al. evaluated the safety and functional effect of NK-cell product derived from HLA partly matched UCB. These NK cells were demonstrated to be well tolerated, and 2 of 4 AML patients became minimal residual disease (MRD) negative in bone marrow after adoptive infusion [108], indicating that immunotherapy based on UCB-derived NK cells has remarkable therapeutic potential. Subsequently, strategies targeting NK cells modification to obtain durable anti-tumor ability experienced great advances. Pluripotent stem cell-derived NK cells engineered with key surface molecules such as high-affinity noncleavable variant of CD16a were demonstrated to improve antibody-dependent cellular cytotoxicity (ADCC) properties of NK cells and contribute to tumor regression in B-cell lymphoma xenograft studies [109, 110]. Clonal NK cell lines containing NK-YS, KHYG-1, NK-92, has become alternative sources for adoptive NK cell therapy [111–113]. Due to potentially broad applicability against tumors and little risk potential for GvHD complications, NK-92 cell line has become a popular agent in thriving clinic trials [114, 115]. However, application of clonal NK cell lines still faces challenges. For instance, irradiation is required before cell infusion to prevent further hyper-proliferation, which in turn drastically limits cell persistence after infusion [116].

### Chimeric antigen receptor NK cell therapy (CAR-NK)

**Fig. 3:**
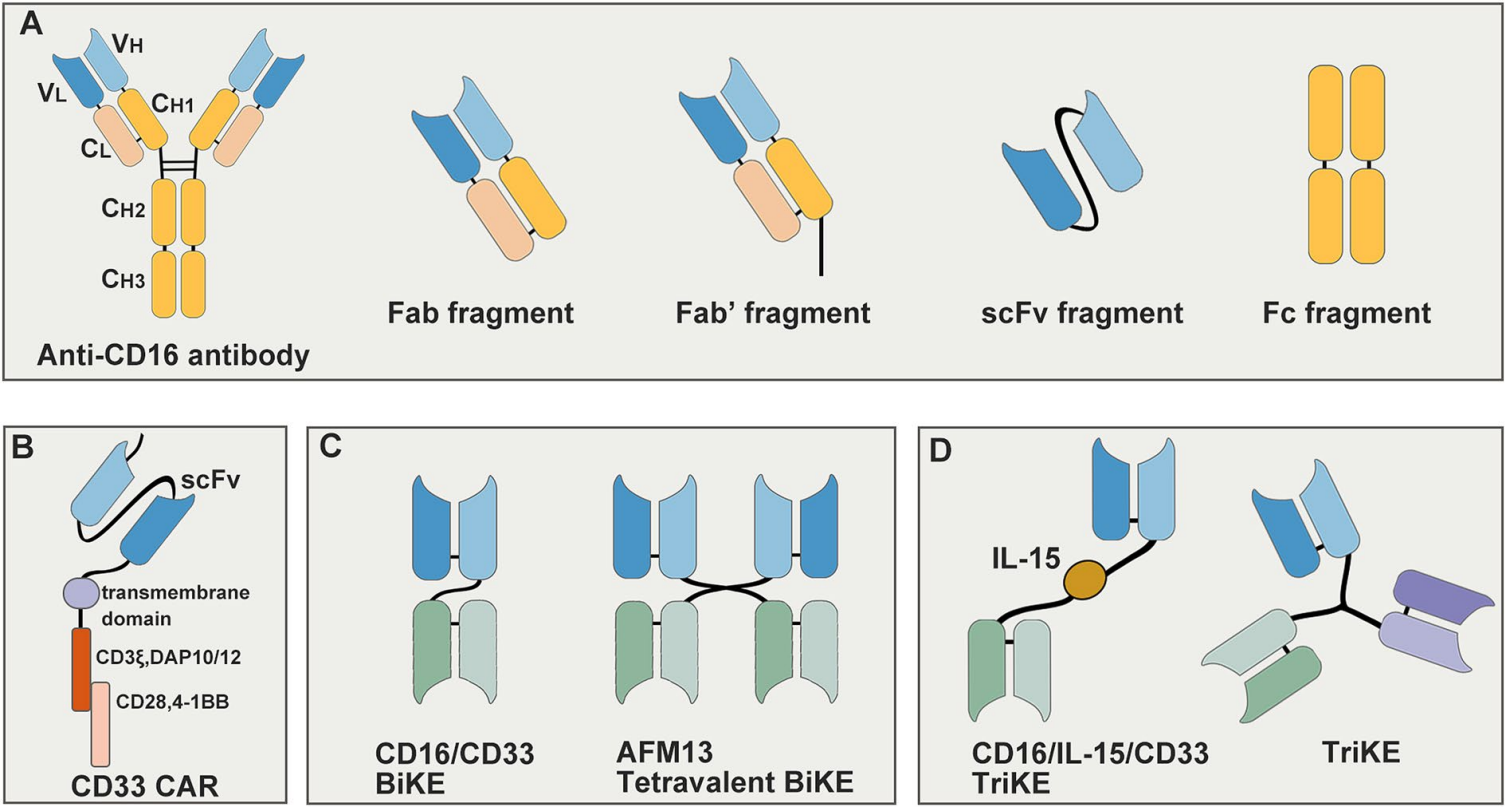
Components and structures of mAb, CAR, BiKE and TriKE.** A** Taking anti-CD16 antibody for example, it can be divided into several components including Fab, Fab’, scFv and Fc. **B** CAR contains an antigen recognition domain, a transmembrane domain and a signal domain providing activating signal to NK cell. As above figure shows, extracellular scFv domain of a CD33 CAR molecule is composed of V_H_ and V_L_ anchored to the transmembrane domain by a flexible hinge, and intracellular part includes two signal domains. **C** A BiKE consists of two scFvs, and a short flexible polypeptide linker joins to prevent dissociation. Take CD16/CD33 BiKE for example, it constructs from V_H_ and V_L_ of anti-CD16 and anti-CD33 antibodies which make it capable of binding both tumor cells and NK cells. AFM is a bispecific, tetravalent chimeric antibody construct that specifically recruits NK cells by two binding sites exclusively for each antigen. **D** Compared to BiKE, CD16/IL-15/CD33 TriKE adds a novel modified human IL-15 crosslinker which can assist in improvement of NK cell cytotoxicity. TriKE can be also designed with a scFv fragment of anti-CD16 mAb and two scFvs of tumor specific antigens. BiKE, bispecific killer cell engager; CAR, chimeric antigen receptor; CD, cluster of differentiation; Fab, fragment of antigen binding; IL-15, interleukin 15; C_H_, constant heavy chain; C_L_, constant light chain; mAb, monoclonal antibody; scFv, single-chain variable fragment; V_H_, variable heavy chain; V_L_, variable light chain; TriKE, trispecific killer cell engage

Clinical achievements and current shortcomings in CAR-T cell therapies consisting neurotoxicity and cytokines release syndrome (CRS) force the improvement of alternative approaches. CAR-NK cells refer to engineered genetically to express specific CAR structures which mainly have three domains: I) extracellular region, containing a single-chain variable fragment (scFv), generally derives from antibodies that recognize surface antigens of tumor cells. II) transmembrane region, anchors CAR to NK cell membrane. III) cytoplasmic domain, transmits activating signals then causes downstream processes and facilitates the killing effects [117–119]. Key components of CAR are shown in Fig. 3. NK cells distinct biology allows them to offer alternative, and perhaps even superior immunotherapeutic strategy in comparison with CAR-T cell therapy. First, CAR-NK cells have reduced risk of GvHD due to a non-HLA-restricted modality [120]. Restricted lifespan of CAR-NK cells in circulation allows no requirement for suicide vectors to prevent excessive expansion [121]. Activated NK cells release GM-CSF and IFN-γ, rather than proinflammatory factors consisting IL-1 and IL-6, implying less likely to occur CRS and neurotoxicity and preferable safety profile [120, 122]. Besides, CAR-NK cells exert more killing modes containing executing cell degranulation, activating apoptotic pathways, and mediating ADCC effects [123]. Cancer-initiating cells (CICs) which play a vital part in tumor recurrence are often characterized by resistant to drugs and irradiation therapy. It has been found that CICs were highly sensitive to NK cells, confirming therapeutic CAR-NK cells could become a strategy for recognition and clearance of CICs and thus prevent tumor recurrence [124]. NK cells have been identified with memory-like function in previous studies, which could allow a more rapid and robust response [125]. Moreover, CAR-NK cells also protect against pathogens such as bacteria and virus, helping to prevent concurrent and secondary infections, which plays an essential role in immunocompromised state in cancer patients.

CAR-NK cell therapies have revealed preliminary potential in a large number of animal experiments [126–128], which laid a sufficient foundation for human studies. For example, arming cytokine-induced NK cells with a neoepitope-specific CAR significantly enhanced their anti-tumor responses and avoided off-target toxicity in AML models [129]. 30 CAR-NK cell related clinical studies for hematologic malignancies were retrieved on https://beta.clinicaltrials.gov [till Feb. 2023]. Most of them are in early-stage aiming to determine the safety and initial efficacy. In a phase I and II clinical trial performed to assess the safety, relative efficacy and overall response rate (ORR), HLA-mismatched cord blood derived anti-CD19 CAR-NK cells were infused to 11 enrolled patients with relapsed/refractory (R/R) CD19^+^ tumors. One of three dose-regimes (1 × 10^5^, 1 × 10^6^, or 1 × 10^7^ cells per kilogram of body weight) of CAR-NK cells were administered after chemotherapies. GvHD, CRS and neurotoxicity were not found after infusion and therapeutic evaluation result was encouraging. Among them, 8 achieved a response including 7 (3 with CLL and 4 with lymphoma) achieved complete response (CR) [NCT03056339] [130]. The shortcoming of this trial was that different therapeutic interventions were received before and after adoptive infusion of CAR-NK cells, exact conclusions regarding the efficacy cannot be drawn. Nkarta, a clinical-stage biotechnology company, announced positive preliminary results of NKX101 and NKX019 in Apr 2022. NKX101, engineered to target NKG2D ligands on cancer cells, showed striking early single-agent activity and no dose-limiting toxicities in R/R AML or myelodysplastic syndromes (MDS) patient populations [NCT04623944]. NKX019 is another leader CAR product engineered to target B-cell antigen CD19. Evaluating NKX019 in B cell malignancies found 3 of 6 patients treated with higher dose level of three-dose regimens achieved 50% CR. It was also proved with satisfying tolerance, the most frequent higher-grade adverse events (AEs) were myelosuppression [NCT05020678]. Although therapeutic potential and clinical outcomes may materially change as patient enrollment continues, progression of these candidates is worthy expecting. Details of other current clinical trials about CAR-NK cell therapy in hematologic tumors are concluded in Table 1.

**Table 1:** Clinical trials of CAR-NK cell therapy in hematologic malignancies ^[1]^

Disease	CAR-NK Product	Targets	NK Cell Sources	NCT Number	Phase	Status	Brief Profile
AML	NKX101	NKG2DL	Allogeneic NK cells	NCT04623944	I	Recruiting	To determine safety and tolerability, cellular kinetics, pharmacodynamics and anti-tumor response. Preliminary results have shown striking early single-agent activity and no dose-limiting toxicities
	NKX019	CD19	Allogeneic NK cells	NCT05020678	I	Recruiting	To evaluate safety and tolerability, cellular kinetics, pharmacodynamics and anti-tumor response. Preliminary results showed that 3 of 6 patients achieved 50% CR
	CAR.70/IL-15 transduced CB-NK cells	CD70	CB-NK cells	NCT05092451	I/II	Recruiting	To determine the safety, efficacy and optimal cell dose
	Anti-CD33 CAR NK cells	CD33	Umbilical cord-NK cells	NCT05008575	I	Recruiting	To assess the safety and efficacy. 6 of 10 patients have received MRD-CR at day 28 assessment. 7 (70%) patients developed grade 1 CRS and only 1 patient developed grade 2 CRS
	NKG2D CAR NK cells	NKG2DL	CB-NK cells	NCT05247957	I	Recruiting	To explore the MTD, clinical safety and efficacy
	Anti-CD33/CLL1 CAR NK cells	CD33	_	NCT05215015	Early I	Recruiting	To evaluate the safety, tolerability, PK, and efficacy. To determine MTD and phase II recommended dose
	Anti-CD33 CAR NK cells	CD33	NK92 cells	NCT02944162	I/II	Recruiting	To determine safety and feasibility
	Anti-CD123 CAR NK cells	CD123	Allogeneic NK cells	NCT05574608	Early I	Recruiting	A dose-escalation study to detect dose-limiting toxicity, incidence of AEs and disease response
	Anti-CD7 CAR NK cells	CD7	Induced pluripotent stem cells	NCT02742727	I/II	Recruiting	To evaluate the safety and efficacy
ALL	CD19-CD28-zeta-2A-iCasp9-IL15-transduced CB-NK cells	CD19 CD28	CB-NK cells	NCT03056339	I/II	Active, not recruiting	To determine the safety and relative efficacy, assess the ORR. Of the 11 patients treated, 8 had a response including 7 (4 with lymphoma and 3 with CLL) had CR and 1 had remission, no major toxic effects were found
	Anti-CD19 CAR NK cells	CD19	_	NCT05410041	I	Recruiting	To observe the safety and efficacy, and preliminarily evaluate the expansion of this product in vivo and the ORR after administration
	Anti-CD19 CAR NK cells	CD19	_	NCT05563545	I	Recruiting	To observe the safety, dose tolerance and pharmacokinetic characteristics
	Anti-CD19 CAR NK cells	CD19	CB-NK cells	NCT04796675	I	Recruiting	To evaluate the primary safety and efficacy
**CLL**	CAR.5/IL15-transduced CB-NK cells	CD5	CB-NK cells	NCT05110742	I/II	Not yet recruiting	To determine the safety, efficacy and optimal cell dose
	CD19-CD28-zeta-2A-iCasp9-IL15-transduced CB-NK cells	CD19 CD28	CB-NK cells	NCT03056339	I/II	Active, not recruiting	To determine the safety and relative efficacy, assess the ORR
	Anti-CD19 CAR NK cells	CD19	_	NCT05410041	I	Recruiting	To observe the safety and efficacy, and preliminarily evaluate the expansion of this product in vivo and the ORR after administration
	Anti-CD19 CAR NK cells	CD19	CB-NK cells	NCT04796675	I	Recruiting	To evaluate the primary safety and efficacy
**MDS**	CAR.70/IL-15 transduced CB-NK cells	CD70	CB-NK cells	NCT05092451	I/II	Recruiting	To determine the safety, efficacy and optimal cell dose
	NKX101	NKG2DL	Allogeneic NK cells	NCT04623944	I	Recruiting	To determine safety and tolerability, cellular kinetics, pharmacodynamics and anti-tumor response
MM	FT576	BCMA	Allogenic NK cells	NCT05182073	I	Recruiting	Dose-escalation study. Till July 2022, no dose-limiting toxicities and no events of any grade of CRS, immune effector cell-associated neurotoxicity syndrome or GvHD were observed among the 9 evaluable patients
	Anti-BCMA CAR NK cells	BCMA	Umbilical or CB-NK cells	NCT05008536	Early I	Recruiting	To assess the safety and feasibility
	Anti-BCMA CAR NK92 cells	BCMA	NK92 cells	NCT03940833	I/II	Unknown status	To assess the safety and feasibility
	Anti-BCMA CAR NK cells	BCMA	_	NCT05652530	Early I	Recruiting	To evaluate the safety and tolerability, and determine the MTD
B Cell Lymphoma	Anti-CD19 CAR NK cells	CD19	CB-NK cells	NCT05472558	I	Recruiting	To assess the safety and efficacy
		CD19	_	NCT05410041	I	Recruiting	To observe the safety and efficacy, and preliminarily evaluate the expansion of this product in vivo and the ORR after administration
		CD19	CB-NK cells	NCT04796675	I	Recruiting	To evaluate the safety and efficacy
		CD19	HLA haploidentical NK cells	NCT04887012	I	Recruiting	To study the safety and efficacy
		CD19	_	NCT04639739	Early I	Not yet recruiting	To manifest the safety and efficacy
		CD19	_	NCT03690310	Early I	Unknown status	To manifest the safety and efficacy
		CD19	_	NCT05570188	I/II	Recruiting	The administration time was 1–7 days after hematopoietic stem cell infusion, to evaluate long-term efficacy and safety
		CD19	_	NCT05645601	I	Recruiting	To investigate the safety and efficacy of JD010 (CAR-NK product)
		CD19	_	NCT05654038	I/II	Recruiting	To evaluate the efficacy and safety
		CD19	_	NCT05673447	Early I	Not yet recruiting	To determine the safety and effectiveness
	FT596	CD19	iPSC-derived NK cells	NCT04245722	I	Terminated	Till 25 June 2021, no dose-limiting toxicities or GvHD were reported. The ORR of whole cohort was 52.9%
	CNTY-101	CD19	Induced pluripotent stem cells	NCT05336409	I	Not yet recruiting	To evaluate the safety, PK, and preliminary efficacy
	CAR.5/IL15-transduced CB-NK cells	CD5	CB-NK cells	NCT05110742	I/II	Not yet recruiting	To determine the safety, efficacy and optimal cell dose
	CAR.70/IL-15 transduced CB-NK cells	CD70	CB-NK cells	NCT05092451	I/II	Recruiting	To determine the safety, efficacy and optimal cell dose
	NKX019	CD19	Allogeneic NK cells	NCT05020678	I	Recruiting	To evaluate safety and tolerability, cellular kinetics, pharmacodynamics and anti-tumor response
	CD19-CD28-zeta-2A-iCasp9-IL15-transduced CB-NK cells	CD19 CD28	CB-NK cells	NCT03056339	I/II	Active, not recruiting	To determine the safety and relative efficacy, assess the ORR
	Anti-CD22 CAR NK Cells	CD22	_	NCT03692767	Early I	Unknown status	To investigate the safety and efficacy
	Anti-CD19/CD22 CAR NK Cells	CD19 CD22	_	NCT03824964	Early I	Unknown status	To investigate the safety and efficacy
	CAR.CD19-CD28-zeta-2A-iCasp9-IL15-transduced CB-NK cells	CD19 CD28	Umbilical or CB-NK cells	NCT03579927	I/II	Withdrawn	To establish the safety and relative efficacy
T Cell Lymphoma	CAR.5/IL15-transduced CB-NK cells	CD5	CB-NK cells	NCT05110742	I/II	Not yet recruiting	To determine the safety, efficacy and optimal cell dose
	Anti-CD7 CAR NK cells	CD7	Induced pluripotent stem cells	NCT02742727	I/II	Recruiting	To evaluate the safety and efficacy

^[1]^Source https://beta.clinicaltrials.gov [till Feb. 2023]

*AEs* adverse events, *ALL* acute lymphocytic leukemia, *AML* acute myeloid leukemia, *BCMA* B cell maturation antigen, *CAR* chimeric antigen receptor, *CB-NK cells* cord blood derived NK cells, *CD* cluster of differentiation, *CLL* chronic lymphocytic leukemia, *CR* complete response, *CRS* cytokine release syndrome, *GvHD* graft versus-host disease, *IL* interleukin, *MDS* myelodysplastic syndromes, MM multiple myeloma, *MTD* maximum tolerated dose, *ORR* objective remission rate, *PK* pharmacokinetics

Further researches are still needed to conquer existing barriers of CAR-NK cell therapy: I) choose more suitable CAR structures, optimize the distance between epitopes and NK cell surface to enhance their effect [131, 132]. II) seek efficient gene transfer approaches. Traditional method for T cells by viral transfection resulted transgene expression of NK cells in low levels and damped their survival [119, 132]. A variety of small molecular compounds have been employed to reduce the repulsion of NK cells to foreign viral particles via charging cells or colocalizing viruses and cells in close proximity [120, 133]. Novel non-viral methods such as electroporation have been also proved to increase transfection efficiency [119]. III) exogenous cytokines are indispensable for survival and proliferation of infused NK cells, while these cytokines cause undesirable AEs like cross stimulating other subpopulation of immunocytes consisting regulatory T cells, which lead immunosuppressive environment for NK cells [134]. Pharmacological interventions, designed CARs targeting NKG2DL-expressing MDSCs and strategies to disrupt TGF-β signaling show the potential to preserve NK cell therapy efficacy [135, 136].

### NK cell-based immune checkpoint blockade

Immune checkpoint receptors anchored on cell surface could mediate the delivery of either inhibitory or activating signals, the balance of which decides if NK cells remain in a quiescent state or kill target cells [40, 137]. Targeting these immune checkpoint receptors has exploited a prospective therapeutic strategy for hematologic tumors. ICB serves the role of reactivating anti-tumor immunoreaction through blocking inhibitory molecules on the surfaces of tumor-infiltrating lymphocytes [138]. Marketing approved and well-studied ICBs of NK cells in recent years were concluded in Table 2.

**Table 2:** Immune-checkpoint blockade of NK cells in hematologic malignancies^[1]^

Targeting Checkpoint Receptor	ICB Product	Clinical Trial	Disease	Phase	Status	Marketing Approved
PD-1	Pembrolizumab (MK-3475)	NCT05514990 NCT05507541 NCT05508867 NCT05493618 NCT05404945 NCT05400876 NCT05355051 NCT05313243 NCT05221645 NCT05204160 NCT05191472 NCT05180097 NCT05179603^[3]^	MM B lymphoma HL MM HL Lymphoma HL T lymphoma DLBCL MM MM HL HL/DLBCL	I/II II III I/II II I/II II II II II II II II	Recruiting Not yet recruiting Recruiting Not yet recruiting Recruiting Recruiting Recruiting Not yet recruiting Recruiting Recruiting Recruiting Recruiting Active, not recruiting	Keytruda, for classical HL and several solid tumors. Initial U.S. Approval: Sep. 2014 Kisplyx. Initial EU. Approval: Aug. 2016 Keytruda. Initial EU. Approval: Jul. 2015 Keytruda, for leukemia, lymphoma and solid tumors. Initial China. Approval: Jul. 2018
	Nivolumab	NCT05385263 NCT05352828 NCT05310591 NCT05272384 NCT05255601 NCT05253495 NCT05211336 NCT05162976^[3]^	B lymphoma HL ALL B lymphoma HL/NHL HL/NHL B lymphoma HL	II I I/II II I/II II I I	Recruiting Recruiting Not yet recruiting Recruiting Recruiting Recruiting Suspended Recruiting	Opdualag, a combination of Nivolumab and Relatlimab, was approved for classical HL. Initial U.S. Approval: Mar. 2022 Opdivo, nivolumab injection, for intravenous use for classical HL. Initial FDA Approval: May. 2016 Opdivo for HL. Initial EU. Approval: Jun. 2015 Opdivo for leukemia and lymphoma. Initial China Approval: Aug. 2019
	Toripalimab (JS-001)	NCT05564806^[3]^	NHL	I	Not yet recruiting	Toripalimab Injection for hematologic malignancies. Initial China Approval: Dec. 2018
	Geptanolimab (GB226)	NCT03639181 NCT03502629 NCT03374007	B lymphoma T lymphoma Lymphoma	II II I	Recruiting Recruiting Recruiting	Not yet
	Nofazinlimab (CS1003)	NCT03809767	Lymphoma	I	Active, not recruiting	Not yet
	SCT-I10A	NCT03821363	Lymphoma	I	Unknown status	Not yet
	Sym021	NCT03311412	Lymphoma	I	Completed	Not yet
PD-L1	Durvalumab (MEDI4736)	NCT05388006 NCT04688151 NCT04462328^[2]^	CLL PCNSL PCNSL	II I I	Recruiting Not yet recruiting Recruiting	Imfinzi, durvalumab injection, for intravenous use for solid tumors. Initial U.S. Approval: May. 2017 Imfinzi for non-small cell lung cancer. Initial U.S. Approval: Feb. 2018 Imfinzi for non-small cell lung cancer. Initial EU. Approval: Sep. 2018 Imfinzi for solid tumors and hematological malignancies. Initial China. Approval: Dec. 2019
	Avelumab	NCT03905135 NCT04328844^[2]^	T lymphoma NHL	I I	Completed Recruiting	Bavencio, avelumab injection, for intravenous use for solid tumors. Initial U.S. Approval: Mar. 2017 Bavencio for neuroendocrine tumors. Initial EU. Approval: Sep. 2017 Not yet for hematologic malignancies
LAG-3	Relatlimab (BMS-986016)	NCT05255601 NCT04913922 NCT04150965 NCT02061761	HL/NHL AML MM Hematologic Neoplasms	I/II II I/II I/II	Recruiting Recruiting Recruiting Completed	Opdualag, a combination of Nivolumab and Relatlimab, was approved for metastatic melanoma. Initial U.S. Approval: Mar. 2022 Opdualag was approved for melanoma. Initial EU. Approval: Sep. 2022 Not yet for hematologic malignancies
	Fianlimab (REGN-3767)	NCT04566978	B lymphoma	Early I	Recruiting	Not yet
	Sym022	NCT03311412	Lymphoma	I	Completed	Not yet
KIRs	Lirilumab	NCT02599649 NCT02481297 NCT02399917 NCT01687387 NCT01592370	MDS Leukemia Leukemia AML MM/NHL	II II II II I/II	Terminated Completed Terminated Completed Active, not recruiting	Not yet
	IPH4102	NCT05321147 NCT03902184 NCT02593045	PTCL T lymphoma CTCL	I II I	Recruiting Recruiting Completed	Not yet
	IPH2101	NCT01248455 NCT01222286 NCT01217203 NCT00999830 NCT00552396	MM MM MM MM MM	II II I II I	Terminated Completed Completed Completed Completed	Not yet
NKG2A	Monalizumab	NCT02921685 NCT02557516	Hematologic malignancies CLL	I I/II	Unknown status Terminated	Not yet
TIM-3	Sabatolimab (MBG-453)	NCT05367401 NCT05201066 NCT04878432 NCT04823624 NCT04812548 NCT04810611 NCT04623216 NCT04266301^[2]^	MDS/AML MDS MDS MDS MDS MDS AML MDS/CML	I/II II II II II I I/II III	Not yet recruiting Not yet recruiting Recruiting Not yet recruiting Active, not recruiting Recruiting Recruiting Active, not recruiting	Not yet
	Sym023	NCT03489343	Lymphoma	I	Completed	Not yet
TIGIT	Tiragolumab	NCT05315713 NCT04045028	NHL MM/NHL	I/II I	Recruiting Recruiting	Not yet
	BMS-986207	NCT04150965	MM	I/II	Recruiting	Not yet

^[1]^Source https://beta.clinicaltrials.gov [till Feb. 2023]. Only approvals from the United States (U.S.), European Union (EU.) and China were recorded in this table

^[2]^Only clinical trials in nearly three years were recorded in this table

^[3]^Only clinical trials in nearly one years were recorded in this table

*ALL*, acute lymphocytic leukemia, *AML*, acute myeloid leukemia, *BCMA*, B cell maturation antigen, *CAR*, chimeric antigen receptor, *CB-NK cells*, cord blood derived NK cells, *CD*, cluster of differentiation, *CLL*, chronic lymphocytic leukemia, *CML*, chronic myeloid leukemia, *CR*, complete response, *CTCL*, cutaneous T cell lymphoma, *DLBCL*, diffuse large B cell lymphoma, *EU*., European Union, *FDA*, the United States Food and Drug Administration, *HL*, Hodgkin lymphoma, *ICB*, immune checkpoint blockade, *IL*, interleukin, *KIRs*, killer cell Ig-like receptors, *LAG-3*, lymphocyte-activation gene 3, *MDS*, myelodysplastic syndromes, MM, multiple myeloma, *NHL*, non-Hodgkin lymphoma, *NKG2A*, natural killer group 2 member A, *PCNSL*, primary central nervous system Lymphoma, *PD-1*, programmed cell death 1, *PD-L1*, programmed cell death ligand 1, PTCL, peripheral T cell lymphoma, *TIGIT*, T cell Ig and ITIM domain, *TIM-3*, T cell immunoglobulin domain and mucin domain-3, *U.S.*, the United States

Targeting PD-1 and PD-L1 mAbs are one of the first research hotspots entering people’s vision, which have been observed to treat both hematologic and solid tumors [139, 140]. Genetic analyses identified that RS cells of Hodgkin Lymphoma (HL) exhibited frequent amplification of 9p24.1, leading to overexpression of correlative gene products PD-L1 and PD-L2. The amplification also increased JAK/STAT pathway in turn by acting on JAK2 locus and further drove PD-L1 expression [140]. PD-L1 overexpression of malignant cells lays the groundwork for strengthening the anti-tumor functions of NK cell via blocking PD-1/PD-L1. Pembrolizumab is a humanized IgG4 PD-1 mAb with high-affinity. Philippe Armand et al. reported Pembrolizumab treatment in 31 HL patients, showing only 5 grade 3 drug-related AEs. CR rate was 16% (5/31) and partial response (PR) rate was 48% (15/31), most responses sustained at least 24 weeks with a median follow-up of 17 months [NCT01953692] [141]. The results identified considerable therapeutic outcome on account of blocking inhibitory immune checkpoints. Moreover, application of PD-1 blockades has broken new ground for advanced R/R tumors. Patients with advanced Sezary syndrome (SS) and mycosis fungoides (MF) suffer worse disease progress and poor OS. One phase II trial found 38% (9/24) ORR including 2 CR and 7 PR among 24 enrolled patients with advanced SS or MF in Pembrolizumab treatment regime of 2 mg/kg every 3 weeks [142]. Pembrolizumab also delineated satisfactory therapeutic effect in other hematologic malignancies such as MM and lymphoma [143, 144].

As above mentioned, KIRs are generally expressed on NK cells to preclude normal cells from damaging. Early study found that KIR epitope incompatible transplants could achieve higher engraftment rates [145]. Lacking of interaction between KIR-MHC I was concluded to trigger NK cell activity, suggesting that specifically blocking the recognition and binding between them could rejuvenate NK cells [146]. Relevant clinical trials are booming, early safety evaluation test of anti-KIRs ICBs draw desirable results but the efficacy tests were inconsistent [147–149]. And addition of KIR blockade improved meaninglessly in the efficacy over single agent PD-1 blockade in some clinical studies [150]. Heterogeneous results may approximately due to that study populations were too small to overcome the biological heterogeneity within and across disease types. Thus, additional benefits accruing from the combination regimens still require longer follow-up time to assess.

Application of ICBs and the resultant breakthrough strategy of taking advantage of immune system for tumor therapy have displayed profound superiorities. However, there are still varieties of challenges need to be addressed. The major barrier is drug-resistant, either lacking of initial response to treatment or with initial promising response but developed resistance during therapeutic stage [151]. Published studies have revealed the minimal expression of PD-1 on NK cells in several tumors, which may lead to primary drug-resistant to ICBs [152]. Strategic approaches such as the combination of immune checkpoint treatments with other therapies including angiogenesis inhibitors and oncolytic viruses have been demonstrated to improve the responses to ICBs [153]. A complex of checks and balances were observed in human immune system that afford response or preserve tolerance. ICBs have the capacity for this homeostatic balance perturbation, causing immune related AEs (irAEs). IrAEs refer to inflammatory adverse events due to non-specific stimulation of immune system by ICBs, generally involving endocrine glands, skin, gastrointestinal tract [154]. IrAEs have been described in many clinical trials including nausea, vomiting, diarrhea, bilirubin increase, rash et al. [NCT01822509] [155]. Besides acute clinical toxicities of these agents, chronic irAEs (usually refer to > 12 weeks sustaining after ICBs discontinuation) are more prevalent [156]. Long-term potential chronic toxicity may be ignored because clinical studies tend to pay attention to the most frequent treatment-associated adverse effects. A generally limited life expectancy for patients with metastatic tumors constrains long follow-up time to exhibit chronic irAEs. Endocrinopathies and rheumatological toxicities has become the most frequent chronic irAEs, and pneumonitis, neuropathy, dermatitis et al. are relatively low-prevalence events [157]. The onset of irAEs varies widely and is hard to predict. Terminating ICBs therapy and beginning high-dose corticosteroids treatment are most commonly used for irAEs control. But there still remains drawbacks including irAEs-overlapped drug toxicities, serious infections, and the risk of suppressing tumor immunosurveillance [158]. Deeper exploration of intrinsic and extrinsic factors that impact ICB response and standard managements built on these hallmarks are intensively needed.

### Cytokine-induced NK cell therapy

Cytokines appear to be critical in many aspects including regulating innate or adaptive immunity, cytogenesis, cell growth, as well as damaged tissue repairment [159]. Earlier results have shown cytokines such as IL-2 could promote the regression of solid tumor models established in animals [160, 161], providing novel insights for augmenting anti-tumor effects of immunocytes containing NK cell by cytokines. Table 3 showed recent applications of cytokine-induced NK cells for treatment of hematological malignancies.

**Table 3:** Recent applications of cytokine-induced and memory-like NK cells for the treatment of hematological malignancies

Methods	Clinical Trial	Phase	Condition or Disease	Intervention/ Treatment	Response	References
Cytokine-induced NK cells	NCT03019666	I	R/R MM, NHL	NK cells cultured ex vivo with IL-15 and nicotinamide (GDA-201)	The overall response rate was 74% in 19 NHL patients,13 had a CR and 1 had a PR	[205]
	_	_	AML	Haploidentical donor NK cells using double immunomagnetic depletion and IL-15 stimulation	Preliminary demonstrated the safety and feasibility of manufactured NK IL15 cells	[206]
	NCT03050216 NCT01898793	II I/II	R/R AML, MDS	IL-15 (ALT-803) activated, haploidentical donor NK cells IL-12 (Aldesleukin) induced NK cells	IL-15 enhanced responder CD8 T cell activation and proliferation, compared with IL-2 alone, demonstrating that additional IL-15 can hasten donor NK cell elimination. These results indicated that stimulating patient CD8 T-cell allo-rejection responses may critically limit allogeneic cellular therapy supported with IL-15	[207]
	_	_	High-risk R/R AML	Double-bright (CD56bright/CD16bright) NK cells from HLA-haploidentical donors modified to express membrane-bound IL-21	Among 13 involved patients, 7 were observed with intermediate or adverse cytogenetics. No dose-limiting toxicities, infusion-related fever, or CRS were observed. OR was 78.6% and CR was 50.0%	[208]
	NCT02763475	II	AML	Haploidentical K562-mb15-41BBL-activated and expanded NK cells administrated with IL-2	The 3-year OS was 83.3% and the cumulative 3-year relapse rate was 28.6%. There were no conclusions regarding efficacy because the study was terminated early	[209]
	NCT01385423 NCT02395822	I II	R/R AML	Intravenous or subcutaneous rhIL-15 after lymphodepleting chemotherapy and haploidentical NK cells	Escalating doses of rhIL-15 (0.3–1.0 ug/kg) were given on 12 consecutive days in a phase 1 trial. Of 26 patients, 36% had robust in vivo NK-cell expansion at day 14, and 32% achieved CR.16 patients received 10 once per day doses of SC rhIL-15 at 2.0 μg/kg on a phase 2 trial. NK-cell expansion at day 14 was seen in 27% of the patients, and 40% achieved remission	[210]
			AML in first CR1 at high risk for recurrence	CTV-1 leukemia cell line lysate-activated NK cells isolated from related HLA-haploidentical donors	2 patients remained relapse-free in post-trial follow-up, exceeding 42.5 months. Donor NK cell microchimerism was detected on day 7 in 10 of 12 patients, with 3 patients having evidence of donor cells on day 14 or later	[211]
	_	_	MDS/AML	IL2-activated haploidentical NK cells	Only transient adverse events were observed in the 16 patients. 6 patients achieved objective responses with CR, marrow CR, or PR	[212]
	_	I	AML	IL-2-dependent NK cell line (NK-92)	None of the involved 7 patients experienced dose-limiting toxicities. Cell dose-dependent effects in the plasma levels of several cytokines were observed	[114]
	_	I	High-risk myeloid malignancies	Membrane-bound IL-21 expanded donor NK cells	Among 13 involved patients, no infusional reactions or dose-limiting toxicities occurred. 1 patient died of nonrelapse mortality, 1 patient relapsed, all others were alive and in remission at last follow-up	[213]
	NCT02477787	II	High-risk AML and MDS	IL-15 and -21-activated NK cells	Intention-to-treat analysis showed a lower disease progression for the NK cell infusion group (30-month cumulative incidence, 35% vs 61%, P = 0.040)	[214]
Memory-like NK cells	NCT03068819	I	Post-HCT relapsed AML	ML NK cells generated by stimulation with IL-12, -15, and -18	4 of 8 evaluable patients achieved CR at day 28. 2 maintained a durable remission for > 3 months, with 1 in remission for > 2 years. No significant toxicity was experienced	[215]
	NCT04024761	I	Myeloid malignancies	Cytokine-induced ML NK cells	In the first 6 enrolled patients, infusion of ML NK cells led to a rapid 10- to 50-fold in vivo expansion that was sustained over months. The infusion was well tolerated, with fever and pancytopenia as the most common adverse events	[216]
	_	_		Cytokine-induced ML NK cells	Clinical responses were observed in 5 of 9 evaluable patients, including 4 CR	[203]
	NCT02782546	II	R/R AML	Cytokine-induced ML NK cells	In 15 patients, donor ML NK cells were well tolerated, and 87% of patients achieved a composite CR at day + 28	[217]

*CR* complete response, *CRS*, cytokine release syndrome, *ML NK cells* memory-like NK cells, *OS* overall survival, *PR* partial response, *rhIL-15* recombinant human IL-15, *RFS* relapse-free survival, *R/R* relapsed or refractory

#### Interleukins

Several interleukin immunotherapies have been permitted by the United States Food and Drug Administration (FDA) such as IL-2 for the treatment of metastatic neoplasms. IL-2 used directly for improving anti-tumor response of NK cell can trace back to 1985, finding that administrating recombinant IL-2 assisted generation of activated lymphokine killer cells [162]. However, there are contradictions between safety and efficacy of IL-2. Low dose can stimulate NK cells expansion, but limited anti-tumor efficacy [163], while high doses enough for efficient anti-tumor response may lead to severe side effects like capillary leak syndrome [164, 165]. IL-2 also assisted to generate and maintain Treg cells to inhibit NK cells via TGF-β and down-regulating surface NKG2D receptors of NK cell [166, 167]. IL-15 was subsequently found to play a critical role in NK cell stimulation with no Treg-mediated immunosuppression. ALT-803 was a super-agonist complex of IL-15, which was evaluated in hematologic malignancies patients relapsed over 2 months, showing well tolerance and remarkable efficacy [168]. IL‐21, with the function of promoting the differentiation and proliferation, increasing IFN-γ production and cytotoxicity of NK cells, is also in exploring process of NK cell-based therapy [169]. A phase I study enrolled 21 patients with B cell malignancies was performed to evaluate the safety, maximum-tolerated dose (MTD) and efficacy of rIL-21 combined with Rituximab, finding toxicities including flu-like symptoms, fatigue, and headache. The MTD was 100 μg/kg and 8 of 19 evaluable patients showed clinical responses after Rituximab-based treatment [170]. This preliminarily confirmed the value of interleukins-based combination treatment regimen.

#### Interferon

Interferons (IFN) are one of the first cytokines to be discovered and have been used therapeutically for decades. The recombinant human IFNs has been approved for treating several tumors such as CML [171], and gradually appeared to be a vital component of combination anti-tumor treatment. Type I IFNs could activate IFN-γ generated of NK cell when combined with IL-12 [172]. Moreover, IFN has proved to be a potential candidate for boosting efficacy of tyrosine kinase inhibitor (TKI) treatment. Mechanism can be concluded that application of TKIs inhibited the degradation of IFN-α receptor on tumor cells, thus increased induction of pro-apoptotic genes and proteins [173, 174]. Combination therapy of IFN-α and Dasatinib in 40 newly diagnosed CML patients demonstrated a steep increase in acceptable tolerability and response rates compared to using Dasatinib only [174]. Engineered interferons in combined treatment regimen have shown promising therapeutic effect in leukemia and lymphoma [174–176], supporting further exploration for potential therapeutic value of IFN.

### NK cell engagers

Bi- and tri-specific antibodies are designed to build efficient immunological synapses between immune cells and malignant cells, specifically recruiting immune cells and forming more densely interconnected to tumor cells. Bispecific antibodies targeting CD3 and specific tumor epitopes to recruit T cells developed rapidly over the past decades. Blinatumomab is one FDA approved CD3/CD19 bispecific T cell engager (BiTE) in Dec. 2014 for adult leukemia. Both CAR T cell therapy and BiTE applications are limited by severe toxicities [177], leading partial study focus more and more turn to NK cell engagers. BiKEs or TrikEs are formed by single variable heavy (V_H_) and light chain (V_L_) of certain antibody, of which a flexible polypeptide linker joined to keep from dissociating [178]. Structures of BiKEs or TrikEs are visualized in Fig. 3. Current NK cell engagers are mainly designed with CD16 and tumor epitopes like CD33 simultaneously, which have several additional advantages compared to mAbs. They are non-immunogenic so that could alleviate many complications of their CAR counterparts [179]. Size of NK cell engagers are small, mainly among 50–80 kDa, which allows efficient tumor penetration and increased biodistribution of the agents. Small size of these fragments allows rapidly elimination through kidneys, contributing to maintain appropriate serum concentration levels and limit associated toxicities [180].

#### BiKE

The engagers targeting CD16 have been chosen in the first generation to trigger NK cell cytotoxicity [181], cooperated with the recognition of different epitopes of tumor cell surface including CD33 or CD33/CD123 on AML cells [182, 183], CD33 on MDS cells [184], CD30 on HL cells [185, 186], CD19/CD20 or HLA-II on B cell lymphomas cells [187, 188]. Dual targeting molecules stand out to recruit NK cells to malignant cells with superior specificity and stronger lysis than traditional mono targeting agents, which extremely attracts further clinical development.

Early back to 1997, F Hartmann et al. had reported a phase I/II clinical research for 15 refractory HL patients with HRS-3/A9, a CD16/CD30 BiKE, finding no explicit dose-side effect till the highest dose administered with 64 mg/m^2^ [185]. AFM13 is a tetravalent chimeric antibody structure that designed to specifically recruit NK cells by combining to CD16A with two binding sites for each epitope but without Fc domain. A dose-escalation trial of AFM13 with administration doses from 0.01 to 7 mg per kilogram of body weight only found mild to medium AEs like headache, nausea, nasopharyngitis, fever, chills and infusion reaction [NCT01221571] [189]. Addition of AFM13 to Pembrolizumab regime in R/R HL patients showed generally well tolerance, and 88% (21/24) ORR at the highest treatment dose [NCT02665650] [190]. Latest research demonstrated that UCB-derived NK cells loaded with AFM13 opening up promising prospects for treatment of RR CD30^+^ lymphoma patients [191]. Clinical trials about AFM13 in patients with other hematologic tumors like T cell lymphoma are in progress [NCT03192202, NCT04074746, NCT04101331].

The platform of BiKE designing is flexible, which enables varieties of alterative components to be assembled. Adding components to BiKE such as scFvs against KIRs, TIGIT, NKG2A and PD-1 receptors provides ability to circumvent inhibitory immune checkpoints therefore drives NK cell associated anti-tumor reactions. Addition of a scFv fragment of TGF-β blocking has also been proved to decrease negative signals in TME [192]. The function of engineered bi-specific antibody combining an anti-CS1 (tumor-specific antigen on MM cells) scFv and an anti-NKG2D scFv was tested in a MM mouse model. The result revealed intensive immune synapse formed between NKG2D^+^ effector cells and CS1^+^ MM cells, promoting NK cells to improve clearance of tumor cells [193].

#### TriKE

Cytokines with activating role such as IL-15, have been cooperated into NK cell engagers to further increase cytotoxicity. When compared with CD16 and CD33 BiKE, addition of IL-15 crosslinker induced superior cytotoxicity, degranulation, and cytokines release of NK cells. It was confirmed that in the immunodeficient mouse model where CD16/IL-15/CD33 TriKE induced maintain and survival of NK cells and exhibited superior anti-tumor effect [194]. C-type lectin domain family 12 member A (CLEC12A) is a specific epitope of AML cells. CD16/IL-15/CLEC12A TriKE was proved to specifically boost proliferation and enhance stimulation of NK cells in vitro experiments. In addition, off-target toxicities were minimized due to absence of CLEC12A on normal cells [195]. Laurent Gauthier et.al reported the generation of tri-functional engagers based on NKp46 and CD16, two activating receptors on NK cell surface, and a tumor-specific surface epitope. This TriKE was more potent in vitro and had similar pharmacokinetics to full IgG antibodies in vivo. It had no off-target effect and effectively inhibited tumor development in the mouse model [196]. They further explored the efficacy of a TriKE targeting NKp46, CD16a on NK cells and CD123 on AML blasts, finding that it had prolonged anti-tumor pharmacodynamic effects and very low inflammatory cytokine induction [197].

NK cell engagers have shown highly efficient anti-tumor functions in vitro and preclinical experiments. It has also provided profound preliminary results that the engagers are safe due to their low possibility of non-specific cytokine release, and their activation only occurs with the presence of tumor cells [198]. However, challenges still remain in this field. Chronic stimulation of NK engager therapies may cause NK cell exhaustion, plausible injection protocols need attempt to limit constant NK cell activation [198]. Antigen-low or -negative malignant cells appear to emerge after killer engagers therapy which could contribute to tumor escape and disease relapse. Thus, additional approaches are required containing combining different engagers and increasing response at lower antigen levels [195]. BiKEs and TriKEs have significant potential for clinical applications with these improved functional characteristics, further explorations are still needed to endow NK cells with superior anti-tumor activities.

### Memory-like NK (ML NK) cells

It was previously believed innate immunocytes consisting NK cells lack antigen-specificity and immunologic memory because their receptor genes cannot undergo somatic rearrangements. However, “ML NK cells” were subsequently observed in mice, NK cells possessing expression of Ly49H receptors had ability to drive the expansion during infectious phase. Expanded effector NK cells then established a pool of long-lived antigen-specific cells with a unique transcriptional signature [199, 200]. NK cells pre-activated through cytokines and then adoptive transferred into vivo showed enhanced proliferation and exhibited restimulation responses to cytokines [201]. Effector function and persistence of syngeneic IL-preactivated NK cells were observed, which also markedly reduced the growth of established mouse tumors [202]. A phase I study observed 5 clinical responses of 9 evaluable AML patients adoptively transferred ML NK cells, demonstrating robust responses of these immunocytes to leukemia cells [203]. An innovative proposal to enhance tumor-specific recognition of ML NK cells by modifying with CARs was come up to increase IFN-γ generation, degranulation and cytotoxicity of NK cells. For instance, ML NK cells derived from lymphoma patients engineered with anti-CD19 CAR reduced lymphoma burden and thus obtained survival improvement in human xenograft models [204]. Han Dong et al. found that arming ML NK cells with a neoepitope-specific CAR significantly enhanced anti-tumor response and avoided off-target toxicity in AML, suggesting that ML NK cells represented a promising cellular platform for modified adoptive cell therapy [129]. CAR-ML NK cells offer apparent advantages including inducing response to NK cell-resistant tumor targets through an obvious synergistic cooperation of CAR-mediated effects and “memory”, representing a powerful tumor immunotherapy approach.

## Conclusions

The roles of NK cell in hematologic malignancies have been revealed in emerging studies, providing scientific basis for novel approaches of immunotherapy. Approaches targeting to combat immunosuppression of NK cells in TME including modifying NK cell with CAR structures or specific engagers, activating NK cell via cytokines or ICBs, inducing ML NK cells, are in different phases of clinical trials and some have been permitted to clinical use already. These immunotherapies have performed encouraging results in safety, persistence and efficiency, especially for patients with tumor relapse and metastasis.

Nevertheless, poor or even no response to these innovative therapies is still remained in a relevant percentage of patients due to dropped expressions of tumor antigen after treatment or complicated immunosuppressive components in TME. Additional explorations are constantly needed to strengthen efficacy of immune therapy based on NK cells. For instance, choose purified, active and low-immunogenic NK cell sources for adoptive infusion to endow superior anti-tumor functions and circumvent detrimental AEs. When designing CAR or engager structure on diverse platforms, it is essential to balance details of different components to ensure high specificity and sensitivity. Moreover, efficacy of combination regimen based on NK cells and other wide-used agents expects for evaluation in more studies. In conclusion, immunotherapy targeting NK cell has become a potential armamentarium and will continually add powerful tools to improve the prognosis of intricate patients with hematologic malignancies in the future.

## Data Availability

Not applicable.

## Abbreviations

ADCC: Antibody-dependent cell mediated cytotoxicity
AEs: Adverse events
ALL: Acute lymphoblastic leukemia
AML: Acute myeloid leukemia
Arg: Arginine
BiKE: Bispecific killer cell engager
BiTE: Bispecific T cell engager
CAR-NK cell: Chimeric antigen receptor natural killer cell
CAR-T cell: Chimeric antigen receptor T cell
CCL: Chemokine CC-chemokine ligand
CD: Cluster of differentiation
CICs: Cancer-initiating cells
CLEC12A: C-type lectin domain family 12 member A
CLL: Chronic lymphocytic leukemia
CML: Chronic myelogenous leukemia
CR: Complete response
CRS: Cytokines release syndrome
CXCR3: C-X-C chemokine receptor 3
Fc: Fragment crystallizable
FDA: The United States Food and Drug Administration
G-CSF: Granulocyte colony-stimulating factor
GM-CSF: Granulocyte–macrophage colony stimulating factor
GvHD: Graft versus-host disease
HLA: Human leukocyte antigen
ICB: Immune checkpoint blockade
IDO: Indoleamine 2,3-dioxygenase
IFN: Interferon
IFN-γ: Interferon-γ
irAEs: Immune related adverse events
KIRs: Killer cell immunoglobulin-like receptors
LDHA: Lactate dehydrogenase A
MCTs: Monocarboxylate transporters
MDS: Myelodysplastic syndromes
MDSC: Bone marrow derived suppressor cell
MF: Mycosis fungoides
MHC: Major histocompatibility complex
MICA: MHC-I chain related protein A
ML NK cell: Memory-like NK cell
MM: Multiple myeloma
MRD: Minimal residual disease
MTD: Maximum-tolerated dose
NADPH: Nicotinamide adenine dinucleotide phosphate
NCR: Natural cytotoxicity receptor
NFAT: Nuclear factor of activated T cell
NK cell: Natural killer cell
NKG2A: Natural killer group 2 member A
NKG2D: Natural killer group 2 member D
NKG2DL: NKG2D ligand
PARP1: Poly-ADP-ribose polymerase 1
PD-1: Programmed cell death protein 1
PD-L1: Programmed cell death ligand 1
PPAR: Peroxisome proliferator-activated receptor
PR: Partial response
ORR: Overall response rate
OS: Overall survival
OXPHOS: Oxidative phosphorylation
ROS: Reactive oxygen species
R/R: Relapsed/refractory
scFv: Single-chain variable fragment
siRNA: Small interfering RNA
SLC1A1: Solute carrier family 1 member 1
SS: Sezary syndrome
TCR: T cell receptor
TIGIT: T cell immunoglobulin and immunoreceptor tyrosine-based inhibitory motif domain
TKI: Tyrosine kinase inhibitor
TME: Tumor microenvironment
TNF: Tumor necrosis factor
TriKE: Trispecific killer cell engager
UCB: Umbilical cord blood
V_H_: Variable heavy chain
V_L_: Variable light chain
